# The Current Landscape of Genetic Testing in Cardiovascular Malformations: Opportunities and Challenges

**DOI:** 10.3389/fcvm.2016.00022

**Published:** 2016-07-25

**Authors:** Benjamin J. Landis, Stephanie M. Ware

**Affiliations:** ^1^Department of Pediatrics, Indiana University School of Medicine, Indianapolis, IN, USA; ^2^Department of Medical and Molecular Genetics, Indiana University School of Medicine, Indianapolis, IN, USA

**Keywords:** genetics, congenital heart disease, phenotyping, next-generation sequencing, phenomics, genomics, mutation

## Abstract

Human cardiovascular malformations (CVMs) frequently have a genetic contribution. Through the application of novel technologies, such as next-generation sequencing, DNA sequence variants associated with CVMs are being identified at a rapid pace. While clinicians are now able to offer testing with NGS gene panels or whole exome sequencing to any patient with a CVM, the interpretation of genetic variation remains problematic. Variable phenotypic expression, reduced penetrance, inconsistent phenotyping methods, and the lack of high-throughput functional testing of variants contribute to these challenges. This article elaborates critical issues that impact the decision to broadly implement clinical molecular genetic testing in CVMs. Major benefits of testing include establishing a genetic diagnosis, facilitating cost-effective screening of family members who may have subclinical disease, predicting recurrence risk in offsprings, enabling early diagnosis and anticipatory management of CV and non-CV disease phenotypes, predicting long-term outcomes, and facilitating the development of novel therapies aimed at disease improvement or prevention. Limitations include financial cost, psychosocial cost, and ambiguity of interpretation of results. Multiplex families and patients with syndromic features are two groups where disease causation could potentially be firmly established. However, these account for the minority of the overall CVM population, and there is increasing recognition that genotypes previously associated with syndromes also exist in patients who lack non-CV findings. In all circumstances, ongoing dialog between cardiologists and clinical geneticists will be needed to accurately interpret genetic testing and improve these patients’ health. This may be most effectively implemented by the creation and support of CV genetics services at centers committed to pursuing testing for patients.

## Introduction

Cardiovascular malformations (CVMs) are the most common birth defects with an incidence estimated at approximately 8/1000 live births (1). Taking into account very high rates of CVMs in spontaneous abortuses, common malformations, such as BAV [present in 1.3% of the population (2)], and latent cardiac diseases, such as aortic dilation, which are not included in the birth incidence of CVMs, genetically mediated CVMs are likely much more common than previously thought. When considering the etiology of CVMs, as opposed to the proportion of CVM cases that manifest as disease at birth, the incidence increases to approximately 5% (1). The common nature of these birth defects, combined with their heterogeneous etiologies, makes genetic evaluation both important and complex.

The underlying causes of CVMs are varied and can include cytogenetic abnormalities, single gene disorders, epigenetic alterations, environmental etiologies, or most commonly, multifactorial etiologies. Chromosomal abnormalities account for 12–14% of all live-born cases and 20–33% of fetal cases (1, 3–5). CVMs can occur as isolated findings, as part of a well-defined syndrome, or in conjunction with additional extracardiac anomalies not formally recognized as a syndrome (6). The American Heart Association has summarized reasons for establishing a genetic diagnosis for cardiac conditions (7). The benefits of a genetic diagnosis include improved longitudinal and acute medical management (8). In addition, a genetic diagnosis allows for the provision of anticipatory guidance, risk stratification for family members, and recurrence risk information (7). Despite an increasing awareness of the genetic basis of CVMs and the clinical importance of making an accurate diagnosis, there remain many questions about the best approach to clinical application of molecular or cytogenetic testing in individuals with a CVM.

Recently, we summarized the overall progress in the molecular genetic analyses of CVMs and current recommendations for clinical application of genetic testing (9). In particular, we reviewed the utility and limitations of chromosomal microarray analysis (CMA) and the emerging clinical roles for whole exome sequencing (WES) and other NGS technologies for CVMs. Here, we focus on the opportunities and challenges of clinical NGS testing and highlight the importance of phenotyping to improve clinical genetic testing interpretation and to drive etiology-centered research. NGS technologies generate abundant amounts of precise human genetic data, but imprecise phenotype data limit the power to determine genotype–phenotype correlation (10). We propose that deep phenotyping of CVMs and existing phenomic analysis methods provide major opportunities for progress analogous to the recently realized efforts in genomics and developmental biology. The integration of genetic findings with deep phenotyping will improve our understanding of disease etiology and advance medical care.

## Epidemiology of CVMs

Cardiovascular malformations represent the single largest cause of infant mortality resulting from birth defects (4). Approximately 25% of infants with CVMs are thought to have syndromic conditions based on the findings of multiple congenital anomalies or neurodevelopmental delays (11). The distinction between syndromic and non-syndromic, or isolated, CVMs can be subtle, and criteria to differentiate these categories are inconsistent between studies. In addition, as genetic diagnostic modalities have become more sophisticated, the spectrum of genetic syndromic conditions has expanded, and therefore earlier assessment of syndromic cases may represent an underestimate.

The high heritability of CVMs provides evidence for an important genetic role in these birth defects. Specific CVMs show strong familial clustering in first-degree relatives, ranging from 3- to 80-fold compared to the prevalence in the population (12). Heritability for some types of CVMs is as high as 70–90%, indicating the strong genetic contribution (13–15). Not all families show evidence of similar types of CVMs, and familial clustering of discordant CVMs has also been documented (16). Because CVMs are so common, the majority of cases occur in individuals without a family history of CVMs despite a high heritability. The prevalence of familial CVM will likely increase as more patients with CVMs survive into adulthood. Epidemiologic studies may underestimate the number of familial cases due to the high rate of miscarriages of fetuses with CVMs and reproductive decisions to limit future pregnancies in families with a child with a CVM.

The sibling or offspring recurrence risk across all types of CVMs is estimated at 1–4%. This empiric recurrence risk suggests that the majority of CVMs have a multifactorial etiology (17, 18). These estimates represent an average of different risks across the population and include individuals with higher recurrence risks due to Mendelian inheritance as well as individuals with lower risks due to a *de novo* event in the affected individual or a teratogenic etiology. Empiric recurrence risks for specific types of CVMs, such as left ventricular outflow tract obstructive defects, are higher. While the incidence of CVMs appear to be similar in most populations, there are some specific types of CVM that show important differences (14, 19, 20). In addition, there is an increased rate of CVMs in populations with increased consanguinity, often attributed to autosomal recessive mutations in disease genes (21–25). Family history of CVMs is one of the most consistently identified risk factors for identifying a CVM prenatally.

## The Genetic Basis of CVMs

Cardiovascular malformations can be subdivided into syndromic and non-syndromic cases. Aneuploidies (disorders of chromosome number) are frequent causes of syndromic CVMs. As genetic testing technologies have evolved, CMA has emerged as a test with higher resolution and increased sensitivity over routine chromosome analysis (i.e., karyotype) for detecting abnormalities. Submicroscopic chromosome deletions and duplications [also known as copy number variants (CNVs)] underlie many genetic syndromes, and the term genomic disorder is used to refer to these conditions. Gene dosage is an important concept underlying CVMs. For many genes, a missing (deletion) or extra (duplication) copy of that gene results in no phenotypic consequences. In contrast, dosage-sensitive genes produce abnormal phenotypes in the absence of two functional genes. 22q11.2 deletion syndrome and Williams–Beuren syndrome are two examples of genomic disorders that are commonly associated with CVMs related to dosage-sensitive genes (*TBX1* and *ELN*, respectively). Variants within *TBX1* and *ELN* are associated with CVMs in non-syndromic patients (26, 27). This fact illustrates an important principle: understanding the genetic basis for syndromic CVMs can identify genes responsible for non-syndromic isolated CVMs.

Because of the increased yield with CMA, it should be the first-line test for genetic analysis in infants with CVMs except in cases that are classic aneuploidies (9). CMA is considered standard of care testing for individuals with developmental disability or multiple congenital anomalies, and it has been shown to be cost-effective (28, 29). Importantly, CNVs have emerged as important causes of both syndromic and non-syndromic CVMs, occurring in approximately 3–25% of syndromic cases and 3–10% of non-syndromic cases (6, 30).

In addition to aneuploidies, chromosome rearrangements, and CNVs as causes of CVMs, mutations at the nucleotide level are also important genetic causes. These mutations are often inherited in a Mendelian fashion, and autosomal dominant, autosomal recessive, and *X*-linked inheritance patterns have been documented for both syndromic and non-syndromic CVMs (31–33). For dominantly inherited conditions, such as Noonan or Holt–Oram syndromes, individual recurrence risks for offspring with the syndrome is 50%. Importantly, not all patients with a particular syndrome have associated heart defects, and the proportion can vary by syndrome. Furthermore, the presence or severity of a CVM in the parent does not predict the severity in the child.

The genetic architecture of CVMs suggests that a majority of non-syndromic cases result from multifactorial causes and behave as a complex trait. Similar to other conditions inherited as a complex trait, isolated CVMs may show familial clustering with reduced penetrance. Nevertheless, Mendelian inheritance does occur, albeit less frequently, and *de novo* mutations are another important cause (34, 35). The distinction between monogenic and complex traits can be overly simplistic, as is drawing a distinct boundary between syndromic and non-syndromic causes. Indeed, variants in genes known to cause syndromic forms of CVMs are now identified in non-syndromic cases. In addition, traits that appear to be monogenic can be influenced by variation in multiple genes, termed modifier genes. The reverse is also true: complex traits can be predominantly influenced by variation in a single gene. These findings likely explain the decreased penetrance and variable expressivity that are so common among both syndromic and non-syndromic CVMs. Currently, there remain many unknowns about the contribution of common variants, rare variants, CNVs, *de novo* mutations, epigenetics, and environmental exposures to the development of CVMs. For these reasons, recurrence risks for apparently isolated CVMs can be difficult to assign, and even in cases of Mendelian inheritance, decreased penetrance and variable expressivity present dilemmas to predicting genetic effect on phenotype. There is need for a systematic approach to accurate and detailed phenotyping in order to begin characterizing these complexities. In addition, these factors are important considerations when contemplating molecular genetic testing in the CVM population.

In an effort to better understand genetic causes of CVMs, systems biology approaches have been used to assess functional convergence of causative CVM genes, effectively combining knowledge of genetics and developmental biology. Interestingly, these approaches have suggested that different CVM risk factors are more likely to act on distinct components of a common functional network than to directly converge on a single genetic or molecular target (36, 37). Developmental pathways acting independently or coordinately contribute to heart development and have been the subject of recent reviews (38, 39). These pathways often exhibit extensive cross talk, and a particular signal can be antithetically regulated at different developmental time points. Systems biology suggests a highly complex milieu in which individual or multiple genetic variants could potentially act to disrupt normal heart morphogenesis. The web of interactions of signaling and transcriptional networks highlighted by these approaches hint at the possibility that some CVMs may result from additive effects of multiple low-effect susceptibility alleles. The integration of genetic analysis with developmental biology knowledge provides a powerful platform for variant interpretation and candidate gene identification, but expanded databases and prediction methods are needed. Improving the assimilation of this information with careful cardiac phenotyping from human studies represents an opportunity to advance our understanding of the etiology of specific CVMs.

## Sequenced-Based Approaches to the Genetics of CVMs

The importance of CNV analysis in both syndromic and non-syndromic CVMs has been documented (6, 9, 40–44). Genetic testing in infants with CVMs is frequently underutilized but indicated in all infants with complex CVMs, except in cases warranting syndrome-specific testing (9, 45, 46). Decisions about additional genetic testing after CMA are less straightforward. The increased sophistication of genetic testing technology provides the ability to interrogate an ever increasing array of genes to identify the molecular basis of disease. Distinguishing testing that has clinical utility is necessary, but few evidence-based guidelines exist, in part, because of difficulties with phenotyping. As a result, clinical experience is the primary criterion utilized in deciding on genetic testing, and substantial practice variation exists for CVMs.

With the development of NGS, large gene sequencing panels have become both technically feasible and cost-effective. As a result, NGS panels for CVMs are developing rapidly. For example, genetic testing for Noonan syndrome has been available for several years, with additional genes being added to NGS panels as they are identified. The current yield of testing using NGS Noonan syndrome panels in suspected cases is approximately 70–85%. As another example, testing for heterotaxy syndrome, situs inversus, and primary ciliary dyskinesia are combined into one NGS panel available from several commercial laboratories.

Several studies have also documented the utility of NGS panels in diagnostic evaluation of CVMs in non-syndromic multiplex families. Blue et al. used a custom NGS panel consisting of 57 genes known to cause CVMs to sequence 16 probands from multiplex families (47). After identifying potential disease-causing variants with the panel in probands, affected family members were tested to confirm segregation with disease. Five variants in 4 genes, *TBX5, TFAB2B, ELN*, and *NOTCH1*, were concluded to be likely disease-causing among the 16 families, giving a diagnostic yield of 31%. A similar study by Jia et al. utilized a slightly different 57 gene panel in 13 multiplex non-syndromic families (48). Altogether, 44 rare variants were identified. After bioinformatics predictions and testing for segregation in other family members, a likely disease-causing variant was established in 6 of 13 families, giving a diagnostic yield of 46%. The causative genes identified in this study (*NOTCH1, TBX5*, and *MYH6*) partially overlapped those of Blue et al. Finally, in a recent study using a panel of 97 genes in 78 unrelated probands with bicuspic aortic valve, 33 potential disease-causing rare variants were identified (49). However, these variants were identified in only 16 of the subjects, indicating that many carried more than one potential disease-causing variant. Because all but two variants were inherited from an unaffected family member, the clinical interpretation of the pathogenicity is difficult. Together, these cases highlight benefits and limitations of NGS panels in non-syndromic patients. First, a substantial number of rare variants will be identified even with relatively small panels. Second, diagnostic yield is high in multiplex families, especially when family members are available for follow-up testing of variant segregation with disease. However, in isolated cases, our current approaches for variant classification and functional prediction make clinical interpretation difficult. Third, careful phenotyping is critical, and distinction of syndromic versus non-syndromic isolated disease is often difficult even in multiplex families. For example, mutations in *TBX5* causes Holt–Oram syndrome, which is characterized by upper limb defects that are highly variable but thought to be completely penetrant with careful examination. In the study by Blue et al. (47), the authors note that subtle hand anomalies may have been missed because radiologic examination was not performed in either family. Finally, while segregation with disease provides strong evidence for pathogenicity of variants, the reduced penetrance of many CVMs suggests that a variant inherited from an unaffected parent does not necessarily rule out disease causation or susceptibility.

Large gene panels have the advantage of increasing the sensitivity of the test, but they also increase the likelihood of identifying variants of uncertain significance (VUS). These increase in direct proportion to the number of genes tested, increasing the complexity of the interpretation and genetic counseling. Importantly, the strength of evidence for disease causality for genes on current panels differs. Some well-established disease-causing genes have a wealth of information about variants, but genes more recently implicated in disease may have much less information available. The latter situation increases the likelihood of finding a VUS. In all cases, it is important for patients to understand that a negative genetic test result does not rule out a genetic cause. The composition of gene panels varies by testing lab. It is critical that the ordering physician understands these factors to order the most appropriate test.

Whole exome sequencing interrogates the coding regions of every gene using an NGS approach. First offered as a clinical genetic test in 2011, the clinical scenarios in which WES is utilized continue to expand. For less than twice the cost of most large targeted gene panels, WES provides sequence data for all known genes, making it comparatively cost-effective. It can be superior to targeted panels for rare syndromes with CVMs in which a genetic cause is suspected but the differential diagnosis is challenging. WES has also been shown to be effective in multiplex families with CVMs. Large, multiplex families with concordant CVMs are good candidates for identifying monogenic disease variants. In addition, recently, a large multiplex family with discordant CVMs across four generations was studied by WES followed by targeted sequencing of candidates (50). A missense variant in *MYH6* was identified in 10 of 11 affected family members and absent in 10 unaffected family members. An additional four unaffected family members also carried the variant. This study not only illustrates the utility of WES for large families but also highlights the complexity of analysis and the challenges that variable expressivity and non-penetrance pose for conclusive interpretation of causality when variants are identified.

Interpretation of causality of a rare variant in a candidate gene is theoretically simplified when the variant occurs *de novo* in the proband. In these cases, the variant is frequently interpreted as likely disease-causing. Therefore, in clinical WES, parental samples are typically requested, if available, in order to aid interpretation. The multisite research study by the Pediatric Cardiac Genomics Consortium provides insight into the frequency of *de novo* variants that are likely disease-causing in a large CVMs cohort (34). Using a trio design to study 362 non-syndromic probands with CVMs, including conotruncal defects, left ventricular outflow defects, and heterotaxy, 249 protein-altering *de novo* variants were identified. Compared with control trios, CVM probands had more *de novo* variants in genes highly expressed during cardiac development and more *de novo* variants with likely damaging effects. The variants were enriched for methylation pathways and were thought to explain approximately 10% of CVMs in the cohort. In a follow-up study of this cohort in which 1213 trios were studied, more *de novo* variants were identified in cases as compared to controls (35). Interestingly, many of these variants were identified in genes known to be important for heart development, and approximately one-third were in genes known to cause syndromic CVMs. Furthermore, there was a striking overlap of variants in genes previously associated with neurodevelopmental delay. These findings may have important clinical impact not only for guiding genetic testing but also for identifying individuals with CVMs who are at increased risk for neurodevelopmental disability and for implementing early intervention.

Limitations to WES in clinical practice include the high likelihood of identifying VUSs, the decreased depth of sequencing as compared to targeted panels, and the increased likelihood of identifying a mutation for a disease unrelated to the clinical presentation or reason for performing the genetic testing. The latter situation mandates pretest genetic counseling to discuss the possibility of secondary or incidental findings. At this time, WES may be the test of choice for syndromic CVMs in which the syndrome is not recognized. It should be considered for both syndromic and apparently non-syndromic CVMs that are inherited in a Mendelian fashion, particularly if the differential is broad or would require multiple targeted panels to test. WES in cases of isolated, non-syndromic CVMs is more controversial due to interpretation ambiguity and financial cost of testing. However, recent data indicate that the incidence of disease-causing *de novo* mutations is high and should prompt consideration of WES especially when parents are available for testing (34, 35).

## The Importance of Phenotyping

As high-throughput technologies, such as NGS, have developed and spread, the volume of genetic data available in clinical and research databases has amassed very quickly. These molecular data are mostly considered to be highly accurate. Accordingly, it is critical that equally accurate phenotype information be used for interpretation of genetic variants. However, the progress in molecular and bioinformatics techniques has vastly outpaced methods to collect and organize detailed and accurate phenotype data across the spectrum of human health and disease (51). Unfortunately, phenotype information associated with genetic diagnoses has not historically been collected and/or reported in a consistent manner. Thus, there is now a pressing need to improve phenotyping practices. The field of phenomics has emerged to address this need, consisting of (1) detailed and accurate phenotype data collection, termed deep phenotyping and (2) computational phenomic analysis (10, 52, 53). With the proper motivation and resources, there is a tortuous but passable route to implement a deep phenotyping approach for clinical testing and etiologic research of CVMs. The following sections describe the current status of phenotype data collection and analysis across the spectrum of human disease, review the current phenotype classification systems commonly used in the clinical care and research of CVMs, highlight the current phenotyping challenges in clinical CVM genetic testing, and emphasize the critical need to harmonize existing phenotype data to advance the field.

## Database Approaches to Deep Phenotyping

Deep phenotyping has been defined as “the precise and comprehensive analysis of phenotypic abnormalities in which the individual components of the phenotype are observed and described” (10). Because there are virtually unlimited ways to describe the phenotype of a patient in the clinical setting, there needs to be a constrained language or set of phenotype definitions to apply systematically in order to analyze differences and similarities between patients. An example of this problem of phenotype unboundedness exists in the Online Mendelian Inheritance in Man database (http://omim.org/), where manually curated phenotype data are highly detailed but unconstrained (54). An ontology is one approach used to organize phenotype data into a structure that is robust for computational analysis. An ontology consists of a set of definitions (or terms) that are assembled as a directed acyclic graph. A number of biomedical ontologies have been developed, including the Gene Ontology, Disease Ontology, Mammalian Phenotype Ontology, and the Human Phenotype Ontology (HPO) (55–58). The HPO is a manually curated ontology that was first developed in 2007 and has since grown to include more than 10,000 terms (each term represents a phenotype definition) (58). The HPO is hierarchically ordered so that the terms at the highest level of the graph consist of the broadest phenotypes. Each term is subdivided into more specific subclass phenotypes until reaching the lowest tier consisting of the most detailed and specific phenotypes. In the HPO, a phenotype term “points” (as a unidirectional edge) to each of its phenotype superclass terms.

In recent years, the HPO has become a heavily used system for phenotyping in the field of human genetics. For instance, the International Standards for Cytogenomic Arrays Consortium was among the first large-scale genotype–phenotype initiatives to adopt the HPO system and demonstrate effectiveness (59). This consortium subsequently became the basis for the Clinical Genome Resource (ClinGen), sponsored by the National Institutes of Health. ClinGen aims to facilitate and establish standards for large collaborative efforts to make genotype–phenotype discoveries and implement these discoveries clinically (60). ClinGen utilizes a public database, ClinVar, as the primary repository of variant and phenotype data. The data are compiled from diverse sources, including domain-specific databases, clinical and research molecular laboratories, clinical providers, and others (61). Similar to the International Standards for Cytogenomic Arrays Consortium, ClinVar utilizes the HPO to define phenotypes and structure data. A number of other databases containing genotype–phenotype data, such as DECIPHER and PhenomeCentral, also utilize the HPO (62, 63).

Whereas the usage of the HPO has increased among genetics providers and investigators, there are many alternative phenotype classification systems in practice. Most of these systems, such as the 10th revision of the International Statistical Classification of Diseases and Related Health Problems (ICD-10), are not designed for the purpose of genetic discovery. Thus, in order to explore genotype–phenotype relationships leveraging separate data sets that potentially contain valuable phenotype information, it is necessary to cross-link systems by mapping phenotypes. These mappings have been created for a number of data sets, but harmonizing databases with different language definitions and structures presents significant challenges and limitations (54). At the very least, the HPO illustrates the motivation and value of establishing a standardized language for deep phenotyping. Importantly, there are user-friendly software applications, such as Phenotips, that enable HPO format data entry (64). Thus, the HPO represents a promising system for phenotyping CVMs in clinical and research settings that would align with many major genotype–phenotype efforts across human disease.

## CVM Nomenclature and Classification Methods

Abbott published the first classification method of CVM phenotypes in the *Atlas of Congenital Cardiac Disease* in 1936 (65). Since then, the precision and accuracy of diagnostic modalities, such as echocardiography and cardiac magnetic resonance imaging, as well as understanding of the morphological and molecular aspects of CV development, have advanced significantly. This has naturally led to the adoption of newer CVM phenotype nomenclatures and classification systems over time. In addition, different practical needs, such as health-care billing, clinical outcomes research, and epidemiology, have given rise to a heterogeneous set of CVM classification systems. One example is the International Pediatric and Congenital Cardiac Code (IPCCC), which was created in 2005 by the International Nomenclature Committee for Pediatric and Congenital Heart Disease. This group includes experts in the fields of pediatric cardiology and cardiothoracic surgery (66). A stated goal of the IPCCC is to facilitate clinical outcomes research across medical centers. Another important CVM phenotyping system, The Fyler Classification System, was created at Boston Children’s Hospital to facilitate local outcomes research and enhance inter-provider clinical communication (67). Since its creation roughly five decades ago, the “Fyler codes” have been expanded and are mapped to the IPCCC. Another frequently used classification system for outcomes research is implemented in the Society of Thoracic Surgeons’ National Congenital Heart Surgery Database (68). Finally, the Botto classification system was developed and tested using data from the National Birth Defects Prevention Study with the principal aim to investigate the etiology of CVMs using epidemiological data (69). Unique among other classification systems, the Botto system emphasized morphological and developmental concepts by grouping individual CVMs into three hierarchical levels. With the recognition that different hypotheses and statistical approaches may require minimum group sizes to achieve adequate power, groupings were also partly based on the known frequency and distribution of individual CVM phenotypes (70). Taken together, there is a long precedent for organizing CVM phenotypes but lack of a consensus nomenclature system. Clinically, this lack of consensus creates barriers in communication with non-cardiac specialists and hinders the attempt to establish genotype–phenotype correlations. In research studies, this lack of consensus creates difficulties comparing results between studies utilizing different classification schemes.

## Obstacles to Deep Phenotyping in CVMs

The lack of a consensus nomenclature system for classifying CVMs can lead to significant confusion and obstacles to the goal of identifying genotype–phenotype relationships. Not only is there a lack of consensus on methods to group CVMs but also many CVMs have synonymous definitions that vary in use across, and in some cases within, clinical programs. For instance, there are at least six synonyms for the diagnosis of perimembranous ventricular septal defect, including infracristal, conoventricular, membranous, paramembranous, and type 1 (71, 72). Furthermore, not all CVMs encountered in patients will fit cleanly into commonly used CVM definitions. For example, a ventricular septal defect is typically defined by the anatomic location of the defect within the ventricular septum, but a defect extending across these anatomic boundaries is not uncommon (e.g., a perimembranous ventricular septal defect that extends into the muscular or inlet portions of the septum). These variations may be developmentally significant. Furthermore, a number of CVMs have morphological subtypes that are classified much differently between systems, such as the Collett and Edwards versus Van Praagh systems, to define subtypes of persistent truncus arteriosus (73, 74). Distinguishing CVM subtypes may improve detection of single gene defects with NGS panels or WES. Taken together, the heterogeneity in routine clinical definition of CVMs is a major impetus for genotype–phenotype databases to utilize a controlled vocabulary structured to manage these intricacies.

There are additional obstacles to consider when organizing data or reports from different clinical programs. For instance, the standard operating procedures in pediatric cardiology imaging laboratories are not uniform across programs despite established recommendations (75). The level of detail provided in clinical reports, such as echocardiography findings, can be variable, and nomenclatures are variable between report-generating software (66). Many aspects of cardiac imaging interpretation depend upon qualitative judgment and experience of the reader, and diagnoses may change or resolve as the patient ages or follow-up studies are performed. Even in circumstances where quantification is feasible, technically standardized, and clinically useful, such as measuring anatomic dimensions, there may be a lack of consensus normative reference databases of healthy children (76). For example, there at least five published normal data sets for calculating *z*-scores of aortic diameters in children (77). Our experience is that calculated *z*-scores range widely depending on the normal data set selected.

With the goal of deep phenotyping in mind, a complete study that includes documentation of negative findings is key to fully defining the patient’s phenotype. However, this may require multiple studies or even different imaging modalities. For instance, in some cases, it can be difficult to absolutely rule in or rule out the presence of extracardiac vascular anomalies, such as abnormal aortic arch sidedness or persistent left superior vena cava with transthoracic echocardiography. Subtle anomalies of coronary artery branching are very difficult to characterize with echocardiography and may not be rigorously interrogated if considered clinically insignificant. Whether these types of subclinical data would advance the understanding of genetics and developmental mechanisms is not known but is quite possible. Additionally, the patient’s age at the time of study may impact not only the technical quality but also the actual diagnoses. Cardiovascular hemodynamics begin to change from the time of birth, cardiac morphology may change as the child grows, and a complete diagnosis may not be reached until normal physiological events, such as closure of the ductus arteriosus. In spite of all of these potential confounders and challenges, the fact that the clinical care of patients is absolutely dependent on accurately characterizing the patient’s phenotype promises to facilitate the implementation of deep phenotyping of CVMs.

## Maximizing the Opportunities for Genotype–Phenotype Correlations

In the field of genetics, there has been important progress in the analysis of phenotype data using computational techniques, sometimes referred to as phenomic analysis. Most phenomic analysis to date has consisted of algorithms used to prioritize lists of candidate disease-causing genes based on phenotype data. Gene prioritization algorithms are useful for interpreting variants identified with NGS techniques, such as clinical WES. The premise for these phenotype-based algorithms is to utilize “semantic similarity,” or the mathematical similarity between a given individual’s phenotype and the phenotypes of reference disease populations, such as those with established genetic disorders. This similarity measure can then be used as the score for prioritizing which variants are most likely to contribute to the individual’s phenotype. Some prediction techniques exclusively utilize phenotype similarity algorithms (78, 79). Alternatively, phenotype-based scores are one component of multidimensional variant prioritization applications that combine algorithms using multiple features, such as the predicted effect of a variant on protein function (80). Variant prioritization applications that incorporate human phenotype data in this manner include Phevor, Phen-Gen, and Exomiser (81–83). There is evidence that incorporation of structured human phenotype data does improve performance (80). Importantly, computational algorithms based on semantic similarity to compare phenotypes across species have also been implemented in applications, such as Exomiser. There is ongoing work to advance phenotype-based computational methods. The accuracy of these methods is likely to improve as more deep phenotyping data are generated and shared.

With the goal of discovering genotype–phenotype relationships for CVMs, the National Heart, Lung, and Blood Institute’s Bench to Bassinet program has generated an unprecedented volume of exome data for patients with CVMs, which have led to major advances toward defining the genetic basis of CVMs (34, 35, 84, 85). This study used a phenotype nomenclature system based on the IPCCC (85). Meanwhile, a large-scale forward genetic screening approach using chemical mutagenesis in mice recently led to novel insights to the mechanisms driving abnormal cardiovascular development (86). Critically, this study undertook a detailed phenotyping approach using fetal echocardiography, postmortem 3D imaging, and histopathological evaluation of unprecedented scale. To illustrate the study’s scope, over 80,000 mouse fetuses were scanned with fetal echocardiography, and over 200 mutant lines with CVMs were identified. The CVMs were classified according to the Mammalian Phenotype Ontology system but were also mapped to human phenotypes using the Fyler codes. The genetic and phenotype data generated from these two large-scale studies present seemingly unbounded opportunities for computational analyses. These include the opportunity to integrate cross-species phenotype data, which will have a key role in advancing understanding of disease pathogenesis (87). These data sets potentially represent the foundation onto which clinical genetic testing data and data from other research enterprises can be added using a uniform phenotyping language. There is the opportunity for the field of CV genetics to harmonize phenotype data with emerging standards used by large genotype–phenotype data sets within the broader field of genomics by mapping to the HPO. Given strong evidence that the genetic basis of non-syndromic CVMs overlaps with neurodevelopmental and other non-cardiac anomalies (35), the integration with other domain-specific genotype–phenotype data sets are likely to produce significant results.

At present, there are clear challenges to implementing the practices of phenomics into routine clinical interpretation of variants and genotype–phenotype research. Some of these challenges are ubiquitous, but others are unique to CVM phenotyping. Most are practical challenges that can be overcome through the efforts of highly motivated clinical and research programs. There is a clear need to adopt a standardized domain-specific CVM nomenclature where individual phenotypes are defined for every patient. Until a uniform nomenclature is adopted, phenotypes will have to be mapped between databases, which pose the risk for error and misclassification (88). On a clinical basis, the established variant databases, such as ClinVar, represent a great opportunity to begin to systematically adopt the reporting of deep phenotyping data. Of equal importance, molecular laboratories should start to require that detailed CVM phenotype data accompany genetic testing requests, which will help force improved clinical practices. These processes will be facilitated if caregivers treating patients with CVMs standardize clinical reporting practices in a manner that is both clinically practical and robust for data analysis. Harmonizing phenotype data across species will facilitate new discoveries. The development of high-throughput, quantitative methods for CVM phenotyping, such as automated digital analysis of imaging data, akin to facial image analysis, may speed discovery by breaking the bottleneck created by the highly specialized, labor-intensive nature of clinical CVM phenotyping (52, 89). While the resources required to advance CVM phenotyping are significant, these will be well worth the added investment to maximize the utility of currently funded genotyping projects. Of equal importance, the clinical interpretation of genetic testing will be improved with deep CVM phenotyping.

## Interpretation of Genetic Testing

The tremendous effort in genomic and phenomic research has a direct effect on clinical testing. Clinical genetic testing moves rapidly to incorporate the most recent research results that have clinical utility and aid patient diagnosis or management. However, because this is an area of rapid accumulation of new data, clinical genetic testing results are not always straightforward since they represent a probability of causing or contributing to disease (90). There are two stages of interpretation of clinical genetic testing results. The clinical laboratory performs the first stage. Variants are classified, compared with ethnic and race-specific information in databases, analyzed using bioinformatic prediction programs, and classified into one of five categories: (1) benign, (2) likely benign, (3) VUS, (4) likely pathogenic, or (5) pathogenic (91). New guidelines have standardized and increased the stringency of interpretation, with more clear criteria for strength of evidence required for interpretation (91). Nevertheless, the interpretations provided for a given variant may differ between clinical genetic testing laboratories. In addition, updates and revisions of the laboratory interpretation may occur as more information is obtained from larger cohorts. For this reason, families should also maintain a relationship with the CV genetics providers, as VUSs often get reclassified over time. A second stage is the interpretation provided by the clinician. Molecular testing results should be one piece of evidence in a diagnostic evaluation. These results need to be interpreted in the context of the patient’s medical history, physical exam findings, disease course, and family history to arrive at a diagnosis. Family history information and the segregation of a potential disease-causing variant within the family may be important information to guide the clinical interpretation of the genetic testing results, especially in cases where novel genetic variants are identified. For CVMs, in which Mendelian inheritance may not be seen or decreased penetrance may make segregation with disease difficult to establish, there are increased challenges to the interpretation of genetic testing results.

**Figure 1:**
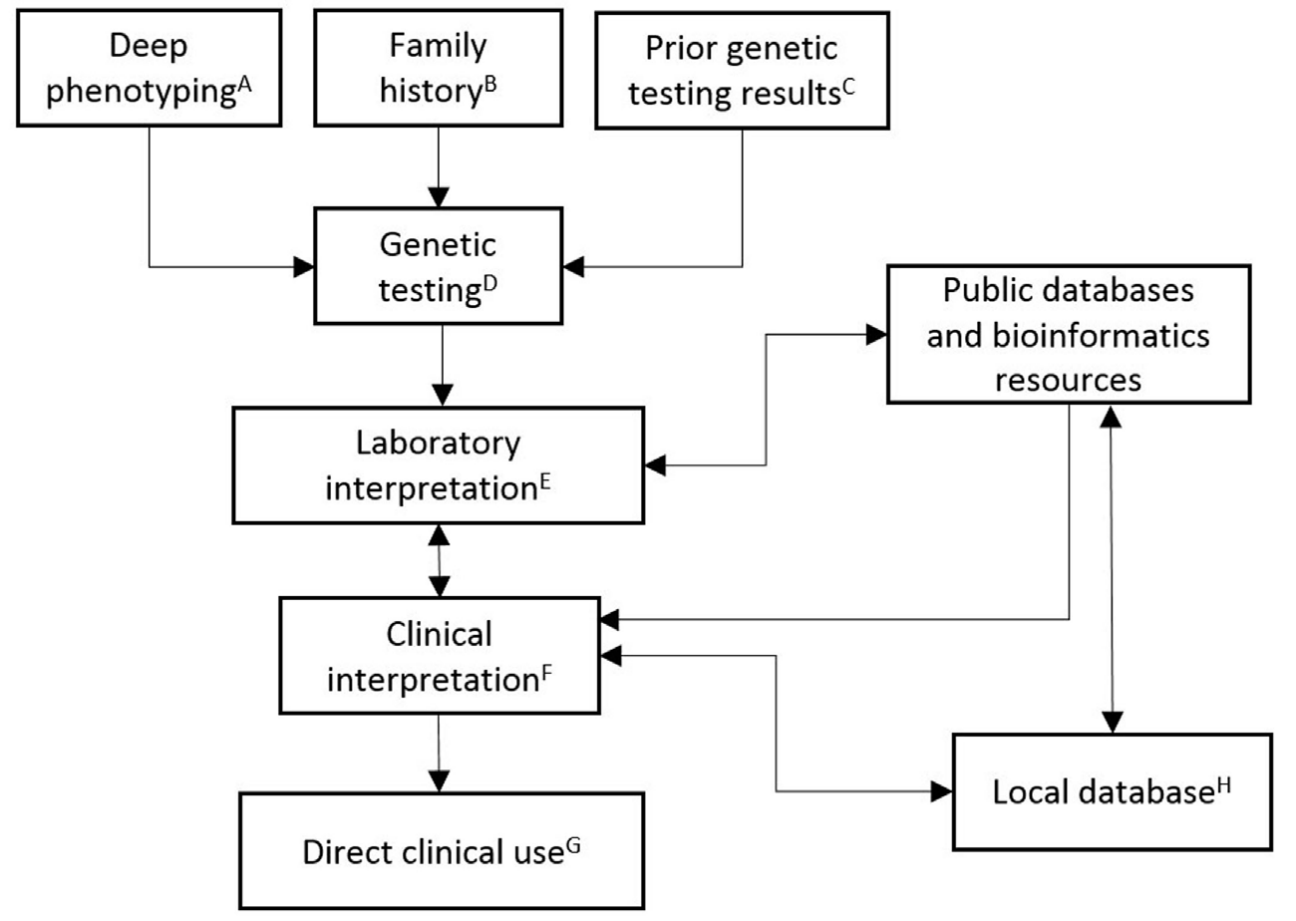
**Schematic outlining the clinical implementation of CVM genetic testing**. **(A)** Deep phenotyping data include complete CVM diagnoses, congenital non-CV malformations, dysmorphic exam findings, neurodevelopmental abnormalities, and other pertinent medical history. Specification of relevant negative findings, including radiographic studies (e.g., head/renal ultrasound), neurodevelopmental evaluation, and specific cardiac evaluations, is important for robust datasets. Age that diagnoses were established or ruled out should be included. Phenotype data should be collected in a structured format (e.g., HPO). **(B)** Family history data are input as a three-generation pedigree, including documentation of relatives with negative cardiac screening. **(C)** Prior genetic testing results data include dates and testing laboratory. **(D)** Genetic testing decisions are patient, family, and disease differential specific. Current clinically available testing options include single gene (e.g., sequencing or deletion/duplication testing), multiple gene (e.g., NGS panels), or genome-wide (e.g., chromosomal analysis, CMA, or whole exome sequencing) testing. **(E)** Laboratory interpretation of genetic testing is based upon ACMG guidelines. High-quality patient data should be provided with the orders for genetic testing. **(F)** Clinical interpretation of genetic testing combines multidisciplinary CV genetics knowledge/expertise with the laboratory interpretation. **(G)** Direct clinical use includes providing results and counseling to family, reporting to health-care providers, recommending treatment, making appropriate subspecialty referrals, making appropriate plan for longitudinal monitoring, and instituting cascade genetic testing and/or family-based cardiac imaging as indicated. **(H)** Local database compiles high-quality phenotype and genotype data for multiple uses, including longitudinal follow up (e.g., completion of cardiac screening in family members or reassessment of variant interpretation), documentation of clinical practices and outcomes, and periodic data harvests for dissemination to public databases and peer-reviewed publication.

A CVM genetic testing workflow begins with the ascertainment of high-quality deep phenotype data (Figure 1). The genetic testing laboratory can improve their interpretation of genetic data when provided with clear phenotype information. The diagnostic interpretation of the clinical care team, longitudinal follow-up and outcome, and family-based clinical information and genetic testing results are all used by the testing laboratory to refine interpretation. Communicating the patient’s phenotype to the testing laboratory or clinical databases, such as ClinVar, is a critical step that is highly susceptible to errors, such as misclassifications or omissions. How can the genetics provider who orders genetic testing communicate the CVM phenotype accurately? The accuracy and completeness of the diagnosis may depend on the sources of the information, which include clinical notes, imaging study reports, procedure notes, or administrative diagnostic codes. The optimal source of this information likely depends on factors specific to patient and medical system. In order to minimize the errors, ideally the genetics provider must have access to all pertinent information (e.g., echocardiography reports, operative reports, cardiac catheterization reports), have sufficient background understanding and experience in CVM diagnoses to accurately define the patient’s CVM phenotype, and have a cardiologist readily available when clarifications are needed. While this process may be effectively conducted by a team of investigators devoted to a specific research project, undoubtedly in most pediatric cardiology centers, there are immense practical challenges to clinically implementing the above scenario for every patient undergoing genetic testing. However, a multidisciplinary CV genetics program consisting of geneticists, cardiologists, genetic counselors, and molecular biologists, which fosters cross-disciplinary education and communication, is actually well suited to meet these needs. These collaborative groups of professionals improve the accuracy of the probabilistic genetic testing information and provide more expertise to the diagnosis and management of the patient.

There remain great opportunities for improving our ability to interpret the results of genetic variation and predicting impact. These are important priorities in all clinical fields that incorporate genetic testing into the diagnosis and management of patients. In the future, identification of genetic modifiers that contribute to phenotypic presentation and explain a portion of the variability and reduced penetrance in these disorders is necessary. This focus will need to include an improvement in our understanding of the impact of rare genetic variation in the population as well as the functional significance of common polymorphisms.

## Summary

In conclusion, there is strong evidence to support CMA testing as a first-line genetic test for infants with clinically significant CVMs. Molecular genetic testing with NGS panels is useful for the evaluation of CVM patients in whom a specific genetic syndrome is suspected. In cases where genetic conditions are highly suspected but a specific syndrome is not recognized, WES may be indicated. NGS panels or WES may be diagnostic in multiplex families with CVMs. Data supporting the potential utility of expanded NGS CVM-gene panels or WES in isolated non-syndromic CVM patients are accumulating, but clinical sensitivity is currently unknown and conclusive variant interpretation remains problematic. Systems biology provides evidence that many CVM genes functionally converge on signaling and transcriptional pathways. Given these considerations, WES or whole genome sequencing will likely ultimately replace NGS panels. However, broader testing will result in ambiguous variant interpretation in CVM patients due in part to variable and expression and reduced penetrance. Incomplete phenotype information and lack of standardized methods for phenotyping also remain significant obstacles. Collaboration between genetics and cardiac care providers and molecular testing laboratories is needed to optimize variant interpretation. There are currently major opportunities to integrate and analyze molecular and phenotype data from human and animal research projects to advance our understanding of the cause of CVMs.

## Author Contributions

The authors (BL and SW) substantially contributed to the conception, drafting, and revising of this article. Both the authors gave final approval of this article to be published and agreed to be accountable for all aspects of the work.

## Conflict of Interest Statement

The authors declare that the research was conducted in the absence of any commercial or financial relationships that could be construed as a potential conflict of interest.

## Abbreviations

CMA, chromosomal microarray analysis; CNV, copy number variant; CVM, cardiovascular malformation; HPO, Human Phenotype Ontology; IPCCC, International Pediatric and Congenital Cardiac Code; NGS, next-generation sequencing; VUS, variant of uncertain significance; WES, whole exome sequencing.
